# Organized Crime and Preventive Justice

**DOI:** 10.1007/s10677-017-9861-7

**Published:** 2018-01-26

**Authors:** Tom Sorell

**Affiliations:** University of Warwick, Coventry, UK

**Keywords:** Serious crime, Preventive justice, Organized crime, Illicit markets

## Abstract

By comparison with the prevention of terrorism, the prevention of acts of organized crime might be thought easier to conceptualize precisely and less controversial to legislate against and police. This impression is correct up to a point, because it is possible to arrive at some general characteristics of organized crime, and because legislation against it is not obviously bedeviled by the risk of violating civil or political rights, as in the case of terrorism. But there is a significant residue of legal, moral and political difficulty: legislation against organized crime is hard to make effective; the harm of organized crime is not uniform, and so some preventive legislation seems too sweeping and potentially unjust. More fundamentally, the scale and rewards of organized crime are often dependent on mass public participation in markets for proscribed goods, which may point to a hidden public consensus in favour of some of what is criminalized. For all of these reasons, I argue that existing preventive policing and legislation against organized crime may be harder to justify than their counterparts in counter-terrorism, at least in the UK.

In liberal jurisdictions criminal justice is primarily backward-looking. For example, a murder has been committed; the culprit is sought and (if all goes well) identified, arrested, fairly tried and convicted, and then proportionately punished. *Preventive* justice is the criminalization of steps that move an agent *forward* toward the commission of a serious crime. The steps may not be particularly harmful in themselves: buying hydrogen peroxide or fertilizer, for example. But individually harmless actions may enact a lethal plan, e.g. to build a bomb capable of killing hundreds. Preventive legislation allows for arrests to be made and charges preferred when there is evidence of (a) a plan to kill hundreds, (b) agreement among the planners on bombing as the means, and (c) procurement by the planners of materials for bomb-making. The case for criminalization in this sort of case is relatively strong, but critics of preventive justice point to other much more problematic offences, such as the glorification of terrorism, or acts of radicalization, or 3rd party offences of non-disclosure of information about a plot.

By comparison with the prevention of terrorism, the prevention of acts of organized crime might be thought easier to conceptualize precisely and less controversial to legislate against and police. This impression is correct up to a point, because it is possible to arrive at some general characteristics of organized crime, and because legislation against it is not currently subject to heated public controversy based on probable violations of civil or political rights, as in the case of terrorism. But there is a significant residue of legal, moral and political difficulty: legislation against organized crime is hard to make effective; the harm of organized crime is not uniform, and so some preventive legislation seems too sweeping and potentially unjust. More fundamentally, the scale and rewards of organized crime are often dependent on mass public participation in markets for proscribed goods, which may point to a hidden public consensus in favour of some of what is criminalized. Preventive policing and legislation in *both* areas, then, are less easily justified than first appears.

The rest of this paper is divided into five sections. I first identify some of the distinctive characteristics of organized crime, which include the maintenance of illicit mass markets, secrecy, ready resort to violence, and willingness to corrupt legitimate officials and politicians. In Section 2 I connect the status of organized crime as serious crime with its tendency not only to do harm to particular individuals, but also to undermine welfare-producing and politically legitimate institutions. In Section 3 I turn to preventive measures, focusing on the Serious Crime Preventive Order (SCPO) in recent legislation in force in England and Wales. I give reasons for doubting the justice of SCPOs, and compare them unfavourably with preventive measures geared to reducing the financial proceeds of crime. I then point out that these latter measures, too, while justifiable, have not proven to be particularly effective. Section 4 goes to the source of a deeper problem with preventive justice in the area of organized crime: namely that there is mass public participation in some of the illicit markets that it depends upon. Does mass public participation in these markets indicate a legitimating consensus in favour of their legalization? Perhaps. But this does not rule out possible mass collusion in the victimization and corruption associated with illicit markets. In order to provide support for decriminalization, market participation needs to take the form of open civil disobedience. Sections 5 and 6 draw conclusions about the distinctive problems of justifying preventive justice in the area of organized crime.

## What is organized crime?

The task of defining organized crime has not proved straightforward. In the US Presidential Commission on Organized Crime in 1986, the difficulties were supposed to be contributed by the term ‘organized’ rather than the term ‘crime’ (Washington Commission 1986: 25). But the fact that some organized crime is often claimed to be victimless,^1^ or to involve illicit markets that should arguably *not* be illicit, makes even the ‘crime’ in ‘organized crime’ problematic. At the same time, the difficulties of capturing in law the kinds of organization that are characteristic of organized crime have not gone away. I begin by trying to identify some general, even if not defining, characteristics.

Organized crime typically consists of co-ordinated illegal activity for financial gain by a group of agents over a significant period of time. The group of agents exhibits a division of labour related to the component specialties involved in e.g. armed robberies or drug importation and dealing. Organized crime excludes more or less spontaneous, short-term looting during a riot involving people who do not know each other, and it may even exclude a one-off-coordinated criminal act (a bank robbery, say) carried out jointly by people who know each other but who will never act together again. The central cases of organized crime addressed in legislation and policy in Western jurisdictions involve more or less enduring crime gangs or families from which criminal plans and acts emanate frequently. Each gang member knows at least some of the other members well, and there are methods of recruitment tied to blood relations or a territory, such as a neighbourhood, or a foreign country of origin.^2^ Of course, the territorial associations of crime gangs do not prevent international operations, notably through the internet, or transnational co-operation (Albanese and Reichel 2014).

Criminal gangs set out to make money by trading in illicit markets, or by robbery, fraud, extortion, kidnapping and murder. These latter, overtly victimizing, forms of organized crime are sometimes directed at ordinary citizens, and sometimes at rival criminals. Typically, the membership and activities of gangs are kept a secret from everyone else, particularly the authorities, and a great deal of ingenuity is directed by gangs at invisibly storing, transporting, and reinvesting the financial proceeds of their primary activities. Violence --sometimes including murder and torture-- is used within gangs to maintain discipline, and against outsiders to make money –through “protection” schemes--or to discourage competition. As for their dealings with the authorities, criminal gangs often try to bribe or blackmail police or judges or politicians for information or influence (Gounev and Ruggiero 2012) and they hire the services of non-criminals (e.g. legitimate lawyers, various kinds of craftsmen, doctors, scientists, accountants) as and when their enterprises or plans require.

A wide variety of crime is engaged in by criminal gangs. The 2010 UK Government Threat Assessment of Organised Crime (SOCA 2010) emphasizes drug importation and trading, immigration crime in various forms, and a wide range of fraud. The shared purpose of all of these forms of organized crime is money-making. Organized crime gangs seek to make themselves rich, or perhaps to make themselves rich and consolidate their power and influence.^3^ What is more, getting rich and gaining power are not typically means to some further organizing end. They are themselves organizing ends. Furthermore, since in many developed jurisdictions convictions for organized crime are now associated with the confiscation of criminal assets, gangs and families must not only find ways of making money but of keeping it in the event that they are caught.

The fact that acquiring and keeping money and power are themselves organizing goals helps to distinguish organized crime from co-ordinated criminal activities conducted to finance terrorism. It is known, for example, that members of the Irish Republican Army carried out at least one huge bank robbery in December 2004 to support its paramilitary activities, and specifically to finance purchases of weapons. More than £20 million in banknotes was taken in an operation facilitated by the violent kidnapping of the family of a bank official (Guardian 2008). If the same amounts could readily have been raised from donations, then the rationale for bank robberies or other conventional crime by the IRA might have seemed slight or non-existent even from its own point of view.^4^ Again, if some hypothetical operation carried out by the IRA had seemed potentially lucrative but involved targeting Northern Irish Catholics, i.e. people from its core political and religious constituency, that, too would presumably have been highly unattractive. For typical criminal gangs, on the other hand, there is no obvious upper limit on the violence used or the targets of it, if there is £20 million to be made. The financial rewards are always a strong consideration in their favour, even a conclusive consideration, if the proceeds and the chances of escaping conviction are big enough.

Criminal gangs aim to maximize profits, and they do so primarily through fraud in legitimate markets or selling goods in illicit markets. To give one example of fraud that is familiar in the USA (Finckenauer 2005: 63-83 and esp. 77), the UK (Telegraph 2013) and elsewhere (Barkhuizen 2014), insurance companies have received large numbers of claims on staged automobile accidents, http://www.telegraph.co.uk/news/uknews/crime/9937896/Police-uncover-countrys-largestcash-for-crash-insurance-scam.html. Other examples of fraud involve the sale over the telephone of worthless land or stock market securities to naïve or vulnerable investors. Then there is cyber crime. Almost everyone who uses email has been on the receiving end of the Nigerian 419 scam. That is, they have received unsolicited emails proposing that, in return for a share, they receive into their bank account a transfer of millions from a foreign jurisdiction.

The involvement of criminal gangs in fraud in legitimate markets is to be distinguished from their activities in illicit markets. For our purposes, two kinds of illicit market can be distinguished, corresponding to what *makes* them illicit. Some markets are illicit in the sense that the legislation of a legitimate government prohibits them. Not every such prohibition of a market is uncontroversial, because the harm done by the goods traded is sometimes unclear, or clear but apparently freely self-inflicted. The harm, if any, done by soft drugs is sometimes thought to be of this type. The harm done by various kinds of alcoholic drink in the US during the Prohibition era might provide a further example. Although marijuana and whisky are open to abuse by buyers, legislation that prohibits trade in them can be thought to be unduly paternalistic, which counts against their criminalization in liberal jurisdictions where they are outlawed.

The other kind of illicit market is where goods are legally or illegally sold but there are strong moral reasons why they should never be. A market in forced labour is a relatively uncontroversial example. Another is a market in entertainment by fights to the death. An intermediate case is where an illicit market in goods and services that are harmless to consumers starts to be infiltrated by criminals with no scruples at all about what they sell. Markets in counterfeit goods are an illustration, because they permit and even invite the sale of dangerous or even lethal products. Cooking oil turns out to be contaminated; counterfeit vodka is discovered to be poison (Guardian 2014); fake branded industrial goods blow up or catch fire; drugs with impurities or in uncontrolled doses start to take lives (Lewis 2009). Since illicit markets have a built-in predisposition to flout even basic trading standards if there is enough money to be made as a result, there is always a danger that merely prohibited markets will tip over into markets that are morally impermissible because of the harmfulness of the goods or services sold.

Neither kind of ground for a market being illicit –legislative prohibition on the one hand or, on the other, the exploitation or harm it involves –necessarily registers with criminal gangs as a reason for not participating in that market. Admittedly, there may be financial reasons for established criminal groups to abide by and perhaps even enforce basic trading standards if a total disregard for these standards would undermine an illicit market. Drugs have to produce a high to attract customers, but they must be safe enough not to make customers regularly ill. In the same way, trafficked women may need to have access to adequate food and health care if they are to attract customers in prostitution. Adequate nourishment and health care are in that sense necessary background standards for the trade in sexual services. But, to labour the point, it is *only* the possible loss of custom and profit –not the badness of harm or exploitation-- that is likely to weigh with criminal gangs in the enforcement of minimal standards. As far as criminal gangs are concerned, there *are* no morally excluded markets or market practices, except perhaps trading in inside information about the activities or plans of criminal groups.

Again, criminal gangs do not operate in illicit markets only. They try to invest the proceeds of their illegal activities into legitimate markets and legitimate businesses. When this happens they employ lawyers to make sure they miss no tax or trading advantages permitted to legitimate businesses. At the same time, they are not above using illegal means, including violence, to gain income from legitimate businesses they do not own or invest in. In local areas they control, criminal gangs or families are often very willing simply to extort money from legitimate businesses. In return for not using violence against the business owners or not destroying their premises, criminal gangs regularly collect a portion of their receipts. Again, typical criminal gangs are very willing to make corrupt payments for exemptions from otherwise successfully enforced regulation or taxation. Through force and pay-offs they are able to increase rewards and reduce costs in legitimate-seeming businesses in which they are involved, that is, businesses that are conducted openly and with apparent regard for legality.^5^

## Why is organized crime serious crime?

Organized crime is often treated in legislation as serious crime. The grounds for treating it this way double as grounds for addressing serious crime with preventive justice measures. For it is natural to think that the more serious the crime, the more its prevention is justified. And if the prevention of a certain variety of serious crime is justified, then so might the criminalization of steps characterisitically leading to its commission. Or at least, criminalization is in order —is in order if—and this is a complex ‘if’—the steps are of a type that really do lead typically to a serious crime, if the penalties associated with taking those steps reliably deter or disrupt criminals, and if they are proportionately lower than the penalties for the fully realized serious crime. These ifs define the work of this section and the next: we shall first give reasons why organized crime should be regarded as serious crime, and then turn to the adequacy of selected preventive justice measures.

In earlier work (Sorell 2016), I have complained of the inadequacy of accounts of serious crime that take into account only directly victimizing offences (Von Hirsch and Jareborg 1991). These are offences in which one person suffers at the hands of another, and where seriousness is a matter of the difference the offence makes to the victim’s standard of life. Murder, which deprives someone of their life, is more serious than burglary, which might deprive someone only of property that they are well able to do without or replace without hardship.

Although accounts along these lines make sense of the relative seriousness of a certain range of offences –assault, homicide and burglary, for example --they do not capture what is wrong with bribery, systemic fraud, misbehaviour in public office and other associated, commonly criminalized activity. In earlier work I made it sufficient for reaching the threshold for serious crime that the organized commission of a type of crime would undermine a welfare-enhancing or harm-reducing institution, and the commission of that crime *was* organized and on an undermining scale. This is the sort of account needed to explain the seriousness or large-scale organized benefit or passport fraud, as well as standard forms of bribery and corruption. It is also the sort of account needed to make sense of the seriousness of common forms of organized crime.

The hybrid account I favour, which makes both undermining welfare-producing institutions and victimizing crime serious crime, is well adapted for explaining why common forms of organized crime are serious. Part of the justification for the fight against organized crime, and long sentences for common specimens of organized crime, must consist of (a) the reasons why victimizing crime is wrong, since the violence and extortion characteristic or organized crime are clearly victimizing. But that cannot be all. The justification must also include (b) reasons why illicit markets are illicit even when market participants are not straightforwardly victims; and (c) reasons why the corruption of officials is wrong. Let  us start with (c).

Criminal gangs operate secretly over years outside the control of a people and its government (Lynch and Phillips 1971: 59); operate for financial gain through violence, fraud and participation in illicit markets; seek exclusive or monopolistic control of an illicit market;^6^ and always stand ready to corrupt officials (Report to the Presidential Commission 1986: 25) That is, they operate out of sight and in defiance of a jurisdiction and sometimes in competition with the authorities in a jurisdiction. This is not because they recognize no authority; on the contrary, they may be quite hierarchical themselves, or acknowledge a pecking order among fellow underworld organizations. The point is that they are a locus of power and loyalty apart from overworld institutions, with secret plans and organization, and with a sense of money-making opportunities unconstrained by either conventional morality or law. They set themselves up as regulatory powers within illicit markets, discouraging new market entrants, intimidating unruly market participants, and acting violently against any would-be rival authorities. They do not merely defy or ignore what the law prohibits, but recruit officials to see that the costs of defiance and disregard for the law in their case are minimized or eliminated.

Organized crime, then, has both victimizing and institution-undermining aspects. Victimizing crime is wrong because of the harm—disablement, loss of life, infliction of pain, fear or other kinds of distress of the person who is attacked or robbed or defrauded. Instead of harm, one can speak in terms of a loss or diminution of standard of life. By contrast, corruption is wrong because, among other things, it works directly against equal treatment before the law, and equal treatment is a requirement of justice. More generally, corruption undermines legal institutions, which have a certain priority in a system of welfare-raising and harm-reducing institutions in developed states. For the police or judges or regulatory bodies to be bought is for access to justice and other forms of welfare to be restricted, or for its distribution to be arbitrary or discriminatory if corrupting agents apply the appropriate influence.

What about (b)? Illicit markets are illicit typically because (i) the products they offer are often harmful; and (ii) sellers in the market know that they are harmful, and consumers don’t know, or are under some sort of compulsion to buy even if they *do* know about the harmful effects. In such cases illicit markets are illicit because they are victimizing. But this is not all. Illicit markets often operate with the collusion of officials, who are paid to turn a blind eye to the victimization. In these cases, the prosecution of illicit market activity is partly justified by what is wrong with victimization and party justified by what is wrong with corruption.

## Preventive legislation in the UK

I have been trying to enlarge on the basis for criminalizing behaviour leading to organized crime: the basis includes the badness of direct, victimizing harm and the badness of undermining socially beneficial institutions by corruption. I now turn to legislation and policy, including preventive legislation and policy, devised to combat organized crime. To make the discussion manageable, I concentrate on the UK.

With the exception of legislation against the intimidation of jurors and witnesses,^7^ and the corruption of police, prison and probation officers,^8^ which acknowledges the steps sometimes taken by organized crime to pre-empt or undermine prosecutions, UK legislation does not dwell on the efforts by organized crime to undermine the justice system or to influence politicians. Instead, it seems to focus on three things: (i) the identification and prosecution of money laundering (Money Laundering Regulations 2007); (ii) confiscating the financial proceeds of crime (Proceeds of Crime Act 2002); and (iii) preventing the resumption by convicted gang members of roles in criminal gangs after they are released (Serious Crime Act 2007).

Although (i) and (ii) are sometimes criticized for irresponsibly and stingily outsourcing judicial or police oversight and investigatory functions to banks (Campbell 2013: 71) I shall concentrate on (iii). This form of UK legislative response to organized crime differs from (i) and (ii) in significantly limiting the liberty of ex offenders. It is associated with the Serious Crime Prevention Order (SCPO) introduced by the Serious Crime Act (2007). Schedule I of this Act recognizes all of the following as serious crimes: drug trafficking; people trafficking; arms trafficking; prostitution and child sex; armed robbery; money laundering; fraud; tax evasion; corruption and bribery; counterfeiting; blackmail; intellectual property offences; environmental offences, including large-scale fishing by prohibited methods, and water pollution. Many of these are directly victimizing. Counterfeiting, intellectual property offences and some environmental crimes (though perhaps not all in Schedule 1) are different. They undermine welfare-producing institutions, or agreements.

The SCPO is one of a very wide range of preventive orders now available to the UK authorities to counteract everything from anti-social behaviour to terrorist activity, broadly construed. Preventive orders in general have been criticized in the academic legal literature for *unduly* limiting the liberty of people (Ashworth et al. 2013), and the question now briefly to be considered is whether this general form of criticism applies to the SCPO (Sorell 2016).

The SCPO enables courts to restrict the activities of those who have been convicted of Schedule 1 offences after they have served their sentences, so as to make it difficult for them to resume their place in a gang or in a network of criminal specialists that career criminals can call upon from time to time. Sections 5 and 6 (3) illustrates ways that SCPOs limit the freedom of ex-offenders:

Examples of prohibitions, restrictions or requirements that may be imposed on individuals (including partners in a partnership) by serious crime prevention orders include prohibitions or restrictions on, or requirements in relation to—

- an individual’s financial, property or business dealings or holdings;
- an individual’s working arrangements;
- the means by which an individual communicates or associates with others, or the persons with whom he communicates or associates;
- the premises to which an individual has access;
- the use of any premises or item by an individual;
- an individual’s travel (whether within the United Kingdom, between the United Kingdom and other places or otherwise).

These are very broad areas of potential interference, and they seem to impose a penal burden on an offender even after a conviction is spent. Serious crime prevention orders can last for 5 years. This is a long time. Instead of returning to society with a clean slate, those who are released from prison after having been convicted of a serious crime are liable to suffer a considerable loss of autonomy over a significant period, with conditions of life significantly determined by judicial decision. Since the threshold for serious crime can be met by someone guilty of no more than online piracy of videos or music, the risk of injustice seems substantial.

To show the SCPO in a more favourable light, it may be necessary to distinguish between one-off and repeat offenders, and, within the category of repeat-offenders, between those who have operated solo, and those who belong to or who regularly co-operate with crime gangs. For obvious reasons, one-off offenders are less credible targets for SCPOs than repeat offenders. As for repeat offenders, the loss of autonomy associated with an SCPO may make sense where there is good evidence that an ex-convict has been involved in organized crime and is likely voluntarily to return to it. Often convicts who have worked with a criminal gang once may face expectations backed by coercion of future co-operation. Convicts leaving prison might face hard-to-refuse demands of collaboration from criminal gangs and might actually be helped *not* to collaborate by serious crime prevention orders.

Let us agree with the critics of preventive orders that the SCPO is a questionable measure when directed at ex-offenders who are *not* members of criminal gangs: are we being too quick to concede that it is appropriate for people who *have* taken part in organized crime? After all, Schedule 1 recognizes a very wide range of organized crime. Do all forms deserve the same treatment? Surely fishing by prohibited methods and intellectual property offences, when carried out by criminal gangs, are not as serious --in the sense of being directly victimizing and inflicting serious harm--as people-trafficking and organized sexual assault of children? Surely the less serious Schedule 1 offences may produce less harm and so justify less well the far-reaching restrictions on liberty which SCPOs may involve?

Here some of the empirical research commissioned by the Home Office in the UK may be relevant. A Home Office Research Report (Francis et al. 2013) on career criminals and organized crime notes that offenders involved in organized crime, while predominantly involved in drug dealing and importation, are not specialists, and that the time they spend in prison deepens and broadens their criminal connections and their knowledge of methods of committing other offences. Nearly a third of all those involved in organized crime covered by the study belonged to the category “versatile and very prolific” --far more than in the other categories included: “mainly violence”, “mixed prolific”, “mainly acquisitive” and “mainly drugs”. Indeed, the “versatile and very prolific” category of organized criminal was twice as large as the next largest “mainly violence” category. Belonging to the “versatile and very prolific” category made it almost certain that in the 5 years leading to arrest for an organized crime offence --an offence with a given term of imprisonment for which someone was co-convicted---the offender had committed some other offence or other, including a .7 probability of having committed violence against a person or a theft and handling stolen goods offence, and a .95 probability of having breached a court order or bail (Francis et al. 2013: 50).^9^ The research also revealed a pattern of increasing seriousness for crimes committed over a career starting in the early teens, with the most serious crimes being committed at around the age of 40.^10^

This research on the career trajectory of people involved in organized crime may help to justify measures that disrupt patterns of association between early career criminals and senior career criminals both during and after imprisonment. This can include disruptions of patterns of association by means of SCPOs. There is also empirical evidence for distinguishing between offences *likely* to be committed by organized crime, and offences that *can* be. Offences likely to be committed by organized crime include forgery and counterfeiting, firearms and drugs offences (Ackerley et al. 2002: Appendix A). All of these are Schedule 1 offences. So if there is evidence that an early career organized criminal is going to associate after imprisonment with known counterfeiters and arms traffickers, that might also be a reason, though admittedly not a decisive reason, for issuing an SCPO banning those associations so as to prevent a career in organized crime.

Although the appropriateness of SCPOs for veterans of very harmful organized crime is at least arguable, we do not have before us a conclusive case in its favour. On the contrary, we have some reasons for thinking that the target group for the imposition of SCPOs is too undifferentiated in the Serious Crime Act (2007). This raises the question of whether other preventive measures against organized crime, measures that at first seem to limit liberty less than SCPOs, might be preferable. For example, might anti-money laundering measures, and powers of confiscating the proceeds of crime be morally superior to the imposition of SCPOs?

Here, too, the answer may be less clear-cut than at first appears. According to an estimate in a 2005 report (Corporation of London 2005), annual proceeds of crime in the UK amount to between £19 and £48bn and only £80 m is confiscated. In the US the corresponding best guess is $110bn, of which £340 m is confiscated (Corporation of London 2005: 16). If these figures are even roughly correct, the effectiveness of measures that are supposed to counteract crime by making it harder for criminals to enjoy the proceeds is low. Since reducing bad effects is part of the moral case for anti-money-laundering measures, the ineffectiveness of those measures matters morally and counts against those measures. The same conclusion seems to be supported by the relatively low number of prosecutions brought by the UK authorities relative to the volume of suspicious activity reports from financial institutions, the relatively small number of banks fined for money-laundering, and the relatively small fines levied up to 2008 when any were levied at all (Harvey 2008).^11^

Again, and perhaps surprisingly, the criminalization of money laundering has come in for criticism on moral grounds. In the US, money laundering can attract a 10-year prison sentence: Douglas Husak objects to this sort of penalty (Husak 2008: 41). According to him, depositing the proceeds of crime X does not add to the wrong of X itself, and only acts that reach the threshold for moral wrongness should be dealt with by hard treatment like imprisonment. Husak would not reject non-penal treatment for money-launderers, say taxing them or making it administratively difficult for them to deposit the proceeds of crime.^12^ His point is that imprisonment is out of order because punishment is out of order for something --an act of depositing money-- that is neither wrong in itself, nor necessarily bad in its effects. His target is US statutes that would punish a criminal for e.g. investing the proceeds of crime into a college fund for his nephew. More generally, his target is the punishment by imprisonment of a very large range of offences that are merely legally prohibited rather than wrong in themselves. He thinks that unbridled criminalization is behind the disproportionately large size of US prison populations, and also the huge and, in his opinion, misdirected investment in the enforcement of US drug legislation (Husak and de Marneffe 2005).

I disagree with Husak’s claim that money-laundering should not be criminalized. Organized crime is a money-making enterprise. To turn its proceeds into money that can safely be kept, criminals need methods of investing it undetected in legitimate businesses. Money-laundering also facilitates the personal enjoyment of the proceeds of organized crime by criminals, and also helps to finance political influence and legislation favourable to industries, such as casinos, hotels and entertainment in which the proceeds of crime have sometimes been invested. Knowingly making it easier through money-laundering for a criminal gang with a long track record of brutality to extend that record, or to diversify their activities to include further offences that are *mala in se,* does seem, in its turn, wrong in itself, and therefore punishable in principle. Money laundering typically finances not only *more* serious harm –an increase in the number of crimes of a given degree of harmfulness-- but an apparatus designed to make harm-production efficient in the future. An apparatus that, so to speak, industrializes crime –that makes the organization of crime more *systematic* and more *efficient*-- is worse than arrangements that simply facilitate *more* crime. The former involves specialization and co-ordination of many people’s efforts. This is different from, and morally worse than, using the proceeds of one’s own crimes to move from a part-time to a full time solo career in burglary.

Admittedly, if a criminal gang had a plan, as in one of the *Godfather* film trilogy, of getting out of organized crime altogether by means of money-laundering in the short-term, the moral case for punishing its money-laundering would have to proceed along different lines from the argument for criminalizing measures that industrialize crime. As things typically are, however, the laundered proceeds of crime stand ready to be used by people whose activities *straddle* the worlds of crime and legitimate business, and who want that straddling to continue. Despite the moral and political exceptionalism of their participation in illicit markets, criminal gangs assert their rights aggressively when they have stakes in legitimate businesses and legitimate markets. Laundered assets -- assets that are easier to keep because they look legitimate and are protected by law --are more attractive for criminal and legitimate business activities *alike* than easy-to-pilfer and incriminating piles of cash in a back room. However, and again as things typically are, there is nothing to make criminals prefer legitimate to criminal activities if the returns of the latter are much greater and the costs or risks are comparable. There is nothing, therefore, to stop laundered assets from financing far more crime than legitimate business when the assets can in principle be ploughed into either. Since criminals typically make deposits, or have deposits made, with a view to financing more crime and an improved crime apparatus, the criminalization of money laundering seems to be perfectly in order.^13^

It may be true that otherwise innocent third parties hired to handle money for laundering are unjustly prosecuted for money-laundering and are only the unwitting associates of criminals. It may be true that it is hard for these third parties to know for certain whether money that they are handling is criminal or not. It may further be true that trying to find out the source of deposits and trying to identify suspicious transactions unfairly imposes financial costs on financial institutions and unfairly transfers legal liabilities to them. It may, finally, be true that when pressed into quasi-forensic roles, employees of financial institutions have discretion to inconvenience and even victimize non-criminal clients that law enforcement officials would not have. In all of these ways, the outsourcing of anti-laundering measures by governments may involve injustice to third parties. These considerations, however, do not count against the criminalization of money-laundering: they are reasons for not delegating responsibility for detecting money-laundering to financial institutions. Perhaps public bodies need to carry out these checks, albeit with money raised from the financial sector.

## Public participation in illicit markets

In the last section I considered some drawbacks of preventive justice measures for organized crime. These seemed either disproportionate, as in the case of SCPOs, or hard to make effective. But there are deeper reasons for thinking twice about organized crime, and these are to do with the way that mass public participation in illicit markets arguably pushes them in the direction of legitimacy.

The main illicit market in the UK is in drugs,^14^ and the drug trade owes its profitability to widespread participation in it by the public. I concede that some participation is grounded in addiction, so that it is not all out “willing” participation. What about the non-addicted? Admittedly, these market participants are not coerced. Still, members of the public who apparently willingly buy services from organized crime have to enter an economic zone that exists in defiance of a jurisdiction that they are otherwise mostly obedient to. This means that even the “willing” participation of people as consumers in illicit markets is conflicted. It requires people to compartmentalize their underworld and overworld activities by keeping e.g. their drug purchases secret from the authorities and other people. Again, the underworld activity of customers in illicit markets seems to defy legislative and other institutions that in their overworld civic roles of voter and juror those people genuinely support. So participation in an illegal market fragments the civic personas of market participants. This makes it quite different from consumer activity in legal markets.

Nevertheless, the fact that people at the receiving end of illicit markets run by crime gangs do not *seem* to be victimized—coerced or harmed against their will—and that there is durable demand for illicit goods and services, makes some non-fraudulent organized crime resemble mainstream business activity. The resemblance sometimes prompts public discussion of the possible legalization of the markets in question. Gambling is one of the formerly illegal activities that has passed into the mainstream of many jurisdictions. The sale of sex and drugs, though legal in some places, is more of a rarity.

Does the fact that there is apparently great public demand for illegal drugs throw doubt on the legitimacy and authority of laws that make the possession, purchase, importation and distribution of drugs illegal? The fact there is a strong analogy between some drugs and some legally sold kinds of alcohol certainly adds to scepticism about the blanket criminalization of drugs. And since only the prevention of *serious* crime justifies liberty-limiting preventive measures, is it reasonable for preventive orders to be issued to veteran drug dealers or drug runners involved only with recreational drugs?

Perhaps the less victimizing the crime and the less harm is done, as in dealing soft drugs as opposed to heroin, the less criminalization in the first place is justified. In the case of small-scale consumers of illegal soft drugs who otherwise do not commit offences, criminalization of possession may be particularly hard to justify. Although the criminalization of possession may be regarded as a preventive measure in that it is meant to discourage transactions in the drug market in general, including the sector involved with the sale of hard drugs, the harm caused by mere possession of any drug, even hard drugs, is surely negligible or non-existent, and the effect on organized crime of confiscating small quantities of soft drugs or of discouraging the smallest players in the market is surely slight. Discouraging drug production, or confiscating big shipments of very harmful because addictive drugs at the point of importation, is surely much more justifiable.

Although these considerations have some force, they do not show that mass participation in drug markets should simply be tolerated by the authorities, or that ordinary consumers of small quantities of non-addictive drugs do nothing wrong by buying regularly from drug dealers. Just because they are otherwise law-abiding, the small-scale consumers we are considering are guilty of exceptionalism. That is, they have pretensions to be their own judges of which laws they will abide by and which they will break. It is true that they are not exceptionalists of the same order as those who direct organized crime, since the latter group are willing to commit violent offences and fraud on a large scale if the profits are high enough and if there is a good chance that they will not be caught. Organized criminals are willing, in other words, to break a whole range of laws, and indeed to direct and even coerce others to do so. Otherwise law-abiding small-scale consumers of drugs are probably much better morally as individuals, and would probably be horrified by the willingness of organized crime to make money from causing harm.

This does not mean that consumers of soft drugs deserve no moral criticism. After all, their purchases foreseeably add to the profit that keeps organized crime in any market, including markets that should always be illicit. Again, the market for soft drugs need not be, and is often intended by criminals not to be, cut off from the market for more profitable and addictive drugs. Although *some* of the illicit drugs sold in the general drug market are probably harmless, many others are not. Being a consumer in a relatively harmless section of an illicit market can expose oneself and others to the promotion of much more harmful drugs. Finally, there are no reliable mechanisms of quality control in an illicit market to ensure that the even the supply of normally harmless drugs does not get contaminated. This fact is a reason why participation in an illicit market for even soft drugs can never be reliably informed, and why, consequently, market participants run a greater risk of harm than consumers in other markets. Or to put it another way, consumers of even the normally harmless drugs in an illicit market can very easily be victimized, and it is a kind of good luck if they are *not* victimized.

There are also considerations about violence. Illicit markets in drugs are regulated by a lot of threatened and actual victimizing violence, some of it life-threatening, mainly inflicted by members of criminal gangs on one another and on customers and dealers who default on payments (Home Office 2007). This means that even if illegal drug purchases are typically uncoerced and do not lead to debilitating addiction, they take place within a market that relies heavily and conspicuously on victimizing violence. Knowledge of that violence is not esoteric: punishments for theft or default are often well advertised to all market participants. Acting as a dealer in the drug market signals a tolerance of that violence. Acting as a regular purchaser can also signal tolerance. What is more, acting as a purchaser can help to fund the purchase of de facto impunity from police investigation and prosecution through bribery. Taking part in the drug market may also contribute indirectly to the recruitment of young people to crime instead of education, and to imbalances in the allocation of policing and other resources in response.^15^ The idea that purchases of soft drugs are normally innocent, then, is doubtful.

Things would stand differently morally if the typical purchase of soft drugs were open and intended as part of a public campaign directed at legalizing drugs. This would elevate drug purchases from acts of self-indulgent exceptionalism to acts of drug consumption seeking legitimization through the political process. But as things actually are, drug purchases by the otherwise law-abiding are carried out quietly and evasively, out of the sight of the same police department that drug purchasers would call in if their houses were burgled or their cars stolen. Perhaps the elimination of this inconsistency or hypocrisy would add to the case for drug legalization. But its existence in the interim certainly counts against citizen participation in drug markets. It does not matter whether the citizen participation is on a large scale or not: the only way to legitimize it is through legislative change, or, short of that, a public campaign of law reform including civil disobedience combined with willingness to submit to arrest for it.

## Prevention of organized crime vs counter-terrorism

It is time to return to the contrast between the fight against organized crime and counter-terrorism. Each is supported by legislation and policy that are eminently criticisable, but criticisable in very different ways. In the case of organized crime, as we have seen, the deepest problems arise from the fact that market activity is at its heart, and that there is large-scale public participation in some of it, sometimes with little resulting harm. Among illicit markets the most problematic are those in which the commodities whose use is criminalized are hardest to distinguish from the commodities traded in legal markets. This is arguably how things stand with the illicit market in soft drugs for recreational purposes, comparable as it is to the entirely legal market in cigarettes and alcohol. It has already been strongly suggested that the right response to the criminalization of participation in questionably illicit markets is political: a consensus can legally be built in favour of law reform by market participants, and in that way some illicit markets can properly be brought out from prohibition.

When it comes to counter-terrorist legislation the deepest problems are quite different. They are not to do with money-making, markets or by the role of big sections of the general public in buying illicit goods. They arise instead from the perceived need to curtail the rights of ideological minorities as a means of preventing violence or attacks by a tiny number within the ideological minority. In the UK, terrorism legislation directly restricts the freedom of association and the freedom of expression, respectively, in as much as membership in certain violence-supporting organizations is outlawed (Terrorism Act 2000), and in as much as the expression of support for acts of terrorism, even in the past, is also an offence. The reason the restriction of the rights of ideological minorities is a *deep* problem, as opposed to a problem simply, is that it seems to relax protections that are constitutive of liberal politics. On the other hand, when the freedom of association and freedom of expression are used to gather and make vocal support for violent political change, those freedoms are arguably being perverted themselves. We might say that both the non-violent support for terrorism and the counterterrorism measures that it inspires denature the liberal democratic context that make them possible. There is no counterpart of this in the fight against organized crime.

Perhaps unsurprisingly, then, preventive justice is radically different in the two cases, and, when questionable, questionable for different reasons. Preventive measures against organized crime in the UK are aimed at reducing its financial rewards and discouraging or disrupting career criminality. *Consumer* participation in criminally organized illicit markets, especially for soft drugs, is treated with a light touch. Depending on the reasons for it, the light touch might be justified. It might be justified if soft drugs used for recreational purposes are relatively harmless. It might be justified if, in addition to being harmless, soft drugs are the subject of broadly based campaigns for legalization and policing of small scale consumption becomes unaggressive to reflect this.

Preventive measures in counter-terrorism have a very different character. Apart from criminalizing the “glorification” of terrorism in broadcasts and print (Terrorism Act 2006: s1), and proscribing Islamist organizations, successive UK governments have introduced many third party offences, including various failures to tell the authorities about the preparation of terrorism. In addition to non-disclosure offences, there has been a devolution of responsibility to non-governmental institutions to monitor local processes of radicalization. For example, universities and prisons have duties under the Prevent strand of UK counter-terrorism policy. These duties are spelt out in the Counter Terrorism and Security Act (2015: s29).^16^

It might be thought that these third-party obligations are the counterparts of anti-money laundering monitoring in financial institutions. But this is a mistake, because some of the new obligations have the effect of clashing with some of the functions of the institutions on which they are imposed. Universities, for example, are among the custodians and channels for the freedom of expression and, up to a point, the freedom of association. Moreover, they are home to *experiments* in free expression. Students at universities have often tried out different kinds of protest tactics, including violent ones. Student newspapers and debating societies have often expanded the limits of free expression and have introduced more variety into the range of discussable political views. The university environment, in short, is a particularly protected space for the exercise of freedoms of expression and thought. Since the line between avant-garde debate and radicalization is sometimes too fine to be discerned, debate is likely increasingly to be suppressed in the name of preventing radicalization. In this way, the new duty under Prevent policy may only help to denature the institutions in the university sector that assume it.

Even *non-violent* extremism is targeted by the *Prevent Duty Guidance for England and Wales* March 2015 (HM Government 2015: para 8). Non-violent extremism includes agitation against “British values,” such as tolerance and democracy. In short, the reinterpretation of terrorism to include much more than violence against civilians to force a change of policy by government introduces a much wider range of possible precursor offences and preventive policing operations. No-one can reasonably object to precursor offences consisting of ordering the ingredients of a bomb or of recruiting personnel for a bomb attack. But recently introduced offences have quite a different character. They call upon institutions to root out practices that are hard to distinguish from things those institutions have tolerated for a long time. What is more, the new duties are being imposed without its being clear that they will be effective in reducing the volume of extremism, violent *or* non-violent. This fact, together with the strong possibility that non-violent extremism is offensive rather than harmful, suggests that criminalization in counter-terrorism has already gone too far.^17^ The case for a comparable conclusion with regard to organized crime is much weaker.

## Conclusion

Preventive justice is not only controversial in counter-terrorism: it is disputable even in the area of organized crime. One reason is that preventive measures in the form of confiscation of assets have not been very effective. But there are also principled objections, based on the connection of organized crime to questionably illicit markets. Some illicit markets trade in products that are arguably no more harmful than certain lawfully traded goods. The comparability of some licit and illicit markets in this respect; and the fact that certain illicit markets are widely patronized despite their being unlawful, provide reasons for legalization when combined with anti-paternalistic arguments. These considerations do not show, however, that all kinds of public participation in questionably illicit markets is legitimate. Participation becomes legitimate when linked publicly to a campaign of law reform. Leaving aside controversially outlawed markets, there is plenty to justify the prevention of organized crime. There is the severe harm it produces, the commodification of this harm, the illegitimate power structures it sustains, and the threat it poses through bribery and coercion to legitimate power structures and the non-criminals they protect. There is also the evidence, at least in the UK, that serious crime occupies people as a career, and that over time criminals diversify their criminal activities. Although Serious Crime Prevention Orders may address these facts in a clumsy way, this does not mean that they are entirely unjustified. In the case of terrorism, on the other hand, preventive justice is controversial because of its multiplication of preparatory offences, its multiplication of 3rd party legal liabilities, and its prohibition of relatively harmless practices that are often innocent exercises of human rights.

## Footnotes

1 See Campbell (2013: 64) ‘Much organised crime does not involve a victim as such, and, as a result, the gathering of evidence may prove problematic...’

2 The United Nations Convention Against Transnational Organized Crime (2001) Article 2 (a) defines an organized criminal group as a group of three or more persons that was not randomly formed; existing for a period of time; acting in concert with the aim of committing at least one crime punishable by at least 4 years’ incarceration; in order to obtain, directly or indirectly, a financial or other material benefit. This definition is unduly permissive, since it allows an ad hoc group formed to carry out a one-off criminal act as a kind of organized crime.

3 Power is emphasised by the 1986 Presidential Commission Report (Washington Commission 1986: 29).

4 This is not to say that terrorists and organized crime gangs cannot make common cause. Kirstin M. Finklea takes this possibility seriously (Finklea 2010: 27-28).

5 Legitimate businesses, admittedly, also resort to pay-offs at times. They are less likely to make direct threats of violence to get their way, however.

6 “From all accounts, organized crime does not merely extend itself broadly, but brooks no competition. It seeks not merely influence, but exclusive influence. In the overworld its counterpart would be not just organized business, but monopoly. And we can apply to it some of the adjectives that are often associated with monopoly-ruthless, unscrupulous, greedy, exploitative, unprincipled.” (Schiller 1971: 73).

7 See (Criminal Justice and Public Order Act 1994), esp. s. 51.

8 The *UK Anti-Corruption Plan* (HM Government 2014) provides for a new offence of police corruption (p. 11), and discusses new powers to target corruption in prisons and post-conviction processes. There is also acknowledgment of corruption in government procurement processes. The UK Independent Police Complaints Commission, in its second report on police corruption, which covers the period 2008-2011, (IPCC 2012) reports relatively few upheld complaints about corruption in the form of perverting the course of justice, but reports surveys suggesting that corruption, is perceived by the public to be widespread.

9 Related Home Office research appears to indicate that convictions for some violent offences is weakly predictive of murder and serious sexual assault. See (Ackerley et al. 2002).

10 Since seniority in a criminal gang comes with the authority to delegate crime to others and so escape arrest and imprisonment oneself, involvement by senior gang members in serious crime is largely missed by the report we are considering. But the existence of crime leaders with authority to delegate crime does help to explain why, at 40, convictions of career criminals decline: some of those criminals have become important enough to direct rather than participate in operations, and to get more junior people involved in the sort of crime that made them eligible for leadership.

11 Fines have increased and have been imposed more frequently in the last 10 years.

12 It is unclear whether Husak would regard a court-ordered confiscation of the proceeds of crime a kind of penal treatment.

13 For different arguments to the same conclusion, see Young (2009). Young partly relies on (Alldridge 2001).

14 Comprehensively described in (Home Office 2007).

15 The argument for treating participation in a violent, illegal market as serious crime may also be an argument for policy that will remove the violence, e.g. by creating a legal and regulated drug market. Discussion of this issue is outside the scope of the present paper.

16 See also the Prevent Duty Guidance in England and Wales (HM Government 2015: para 114) “Universities will be expected to carry out a risk assessment for their institution which assesses where and how their students might be at risk of being drawn into terrorism.

17 The Behavioural Sciences Unit of MI5 (the UK security service addressing domestic threats) distinguishes between “talkers” (whose extremism is registered in speech and writing only) and “walkers”, who are willing to kill or injure others. This is an important distinction in the justification of preparatory offences: criminalization needs to fasten on the “walkers”.
